# Endogenous microRNA clusters outperform chimeric sequence clusters in Chinese hamster ovary cells

**DOI:** 10.1002/biot.201300216

**Published:** 2014-02-12

**Authors:** Gerald Klanert, Vaibhav Jadhav, Konstantina Chanoumidou, Johannes Grillari, Nicole Borth, Matthias Hackl

**Affiliations:** 1 Department of BiotechnologyBoku University Vienna, Austria; 2 ACIB GmbH, Austrian Centre of Industrial BiotechnologyGraz, Austria

**Keywords:** Chimeric sequence, CHO cell, Endogenous miRNA, MicroRNA engineering, MiRNA cluster

## Abstract

MicroRNAs (miRNAs) are small non-coding RNAs (∼22 nucleotides) which regulate gene expression by silencing mRNA translation. MiRNAs are transcribed as long primary transcripts, which are enzymatically processed by Drosha/Dgcr8, in the nucleus, and by Dicer in the cytoplasm, into mature miRNAs. The importance of miRNAs for coordinated gene expression is commonly accepted. Consequentially, there is a growing interest in the application of miRNAs to improve phenotypes of mammalian cell factories such as Chinese hamster ovary (CHO) cells. Few studies have reported the targeted over-expression of miRNAs in CHO cells using vector-based systems. These approaches were hampered by limited sequence availability, and required the design of “chimeric” miRNA genes, consisting of the mature CHO miRNA sequence encompassed by murine flanking and loop sequences. Here we show that the substitution of chimeric sequences with CHO-specific sequences for expression of miRNA clusters yields significantly higher expression levels of the mature miRNA in the case of miR–221/222 and miR–15b/16. Our data suggest that the Drosha/Dgcr8-mediated excision from primary transcripts is reduced for chimeric miRNA sequences compared to the endogenous sequence. Overall, this study provides important guidelines for the targeted over-expression of clustered miRNAs in CHO cells.

See accompanying commentary by Baik and Lee DOI: 10.1002/biot.201300503

## 1 Introduction

The advantage of Chinese hamster ovary (CHO) cells over microbial production systems is that they can produce proteins with human-like post translational modifications [1]. Yet the space/time yield of recombinant proteins produced in CHO cells is at least ten–fold lower when compared to microbial hosts [2]. Different bioprocess [3–5] and medium optimizations [2, 6, 7] were developed and implemented to overcome this drawback. Another approach has been to directly improve the host cell by genetically engineering cellular functions such as apoptosis [8–11], productivity [12–14], and metabolism [15–17]. Given the wealth of published data in this field, the references given above are illustrative of the strategies employed, but not an exhaustive survey of the literature. In this context, microRNAs (miRNAs) are increasingly considered as promising tools for CHO cell line development as they were shown to be essential regulators of cellular functions that support cell cycle progression and protein expression (for example [18–20]).

The biogenesis of this class of small non-coding RNAs, with a length of approximately 22 nucleotides, is a complex multi-step process that relies on coordinated action of several enzymes and RNA binding proteins. First, primary miRNA transcripts (pri-miRNAs), which are called miRNA clusters when they give rise to more than one mature miRNA, are long single-stranded RNA molecules that are usually generated by RNA polymerase II or occasionally by RNA polymerase III. Pri-miRNAs from intergenic regions are processed by the Drosha/DGCR8 protein complex, which cleaves the RNA to form 50–70 nt long RNAs exhibiting a characteristic RNA-secondary structure consisting of a dsRNA region connected by a short loop sequence. These intermediate forms of miRNAs are termed precursor-miRNAs (pre-miRNAs), but are often referred to as “hairpins” or “stem-loops”. The hairpins are exported into the cytoplasm where the RNase-III enzyme Dicer catalyses the production of two largely complementary mature miRNAs that form a duplex. One or sometimes even both strands are selectively incorporated into the RISC complex and used as guides to scan for mRNAs with complementary sequences. Once a target is bound to the protein-miRNA complex, it is either degraded or translationally repressed [20–24]. Despite the small size and principal ease of over-expression of miRNAs, their biogenesis mechanism is complex, requiring well characterized tools to achieve stable over-expression [25, 26] or knockdown in mammalian cells [27, 28].

With respect to CHO cells, the identification and annotation of the miRNA transcriptome [29, 30] allowed the use of mature endogenous miRNA sequences (CHO-sequences in contrast to orthologous sequences from human, mouse, or rat) to study their biological effect. These gain-of-function studies employed either transfection of synthetic mature miRNA mimics [31], or plasmid encoded pre-miRNAs, that were pieced together from mature CHO miRNAs and ectopic flanking and loop sequences from mouse (“artificial chimeric miRNA construct”) [32]. These gain-of-function studies needed no information on the genomic location or hairpin structure of miRNAs and could be rapidly performed using DNA synthesis. As this technology had been developed for construction of short hairpin (shRNA) for gene knockdown in a variety of cellular systems, its use for miRNA engineering in CHO was an obvious choice [26]. Soon after the publication of the CHO genome in 2011 [33], pre-miRNA sequences and the respective genomic loci were published [34], making it possible to amplify and clone endogenous pri–miRNAs and to use them for cell line engineering (“endogenous miRNA construct”).

In the following study we compare both constructs for the expression of two different miRNA clusters, miR-15b-16 and miR-221-222. Our data clearly indicate that endogenous miRNA constructs are better suited for expression of miRNA clusters than artificial constructs.

## 2 Material and methods

### 2.1 Cell culture

A previously described recombinant serum- and L-glutamine-free suspension production cell line CHO DUKXB11 EpoFc 14F2 [35, 36] was cultivated in CD CHO medium (Gibco®, Carlsbad, CA, USA) supplemented with 0.19 μM Methotrexate and 0.2% Anti-Clumping Agent (Gibco) in a shaker-incubator at 37°C, 7% CO_2_ and 140 rpm.

### 2.2 Genomic DNA isolation

gDNA was isolated using the DNeasy® Blood & Tissue Kit (Qiagen, Germany) according to the manufacturer's protocol. In brief, 5 × 10^6^ cells were harvested and resuspended in DPBS no calcium, no magnesium (PAA, Austria) including proteinase K. Buffer AL was added and the samples were incubated at 56°C for 10 minutes. Ethanol was added and the suspension was filtered through the DNeasy mini spin column by centrifugation. After washing with Buffer AW1 and AW2, the membrane was dried and the DNA was resuspended by the addition of 200 μL Buffer AE followed by a centrifugation step. The quality and quantity of the gDNA were determined by UV-VIS spectrophotometry (Nanodrop ND–1000 spectrophotometer, Thermo Scientific Inc., Waltham, MA, USA).

### 2.3 Cloning of miRNA cluster expression plasmids

**Figure 1:**
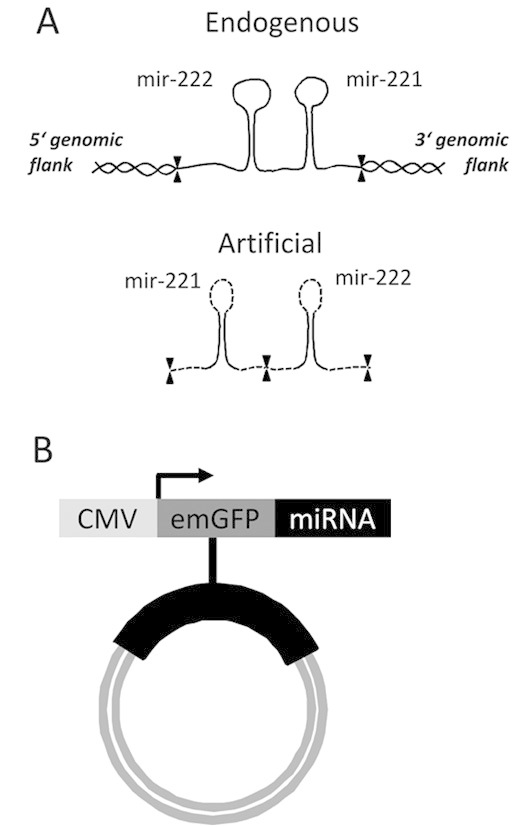
Schematic representation of endogenous and artificial constructs for over-expression of microRNA clusters. **(A)** Endogenous mir-221/222 was PCR amplified from CHO-K1 genome, using primers 70 nt up and downstream of the genomic location. Primers contained restriction sites, which were used for cloning the sequence into a pcDNA 6.2 expression vector containing emGFP. Artificial constructs of ∼60 nucleotides are composed of CHO-specific mature miRNA sequences (solid lines) as well as the flanking and loop sequences of mir-155 (dotted lines). Artificial mir-221 and mir-222 were synthesized individually and cloned into the pcDNA 6.2 vector using restriction sites as indicated by black arrows. **(B)** A schematic of the pcDNA 6.2 expression vector used in this study, with CMV-controlled emGFP expression and microRNA cloning site contained in the 3'UTR of emGFP.

The chimeric miR-15b/16-2 and miR-221/222 clusters were created by concatenation of miRNA expression plasmids with artificial miRNA constructs (Fig. 1) as previously described [26, 32]. In short, the chimeric miRNAs, consisting of the mature CHO miRNA sequences with restriction sites on either end, and an optimized murine loop sequence (Supporting information, Table 1), were cloned into the 3' untranslated region (3'UTR) of emerald green fluorescent protein (emGFP) located in the pcDNA6.2–GW/EmGFP–miR vector (BLOCK–i™ Pol II miR RNAi Expression Vector Kit, Invitrogen Inc., Carlsbad, CA, USA), already containing artificial flanking regions. One of the two corresponding chimeric cluster miRNAs was cut out, including the artificial flanking regions, and inserted into the plasmid with the other chimeric cluster miRNA for artificial cluster generation (Fig. 1) according to the manufacturer's instructions.

For endogenous miRNA-cluster construct generation, the relevant gDNA regions were amplified by polymerase chain reaction (PCR) using primers located in the flanking regions at least 20 bp from the outermost miRNAs of each cluster (Fig. 1A, Supporting information, Table S1). The resulting PCR products were cloned into the same region of the pcDNA6–GW/EmGFP–miR vector (Fig. 1B), and the accuracy of the insertion and the sequence were confirmed by conventional sequencing.

### 2.4 Transfection

Nucleofection was performed using the Amaxa Nucleofector I/program H–14 and the Amaxa cell line nucleofector kit V (Lonza Group Ltd., Switzerland). 10^7^ cells in exponential growth phase were harvested and resuspended in 82 μL of Cell Nucleofection Solution V supplemented with 18 μL supplement I and 10 μg of the respective endotoxin-free plasmid. The same plasmid without insert was used as negative control. The solution mixtures were transferred into a cuvette and nucleofected. After transfection, 2 mL of pre-warmed media was added to the cuvette and the whole solution was transferred into a 125-mL shaking flask (Corning®, Life Sciences, Tewksbury, MA, USA) containing 58 mL of pre-warmed media. Immediately after the transfer, the cells were divided into 2 × 30 mL aliquots generating two technical replicates. Cells were incubated for 2 hours at 37°C, 7% CO_2_ and humidified air without shaking for recovery. Subsequently, culture flasks were transferred into the shaking incubator at 37°C, humidified air containing 7% CO_2_ and constant shaking at 140 rpm.

### 2.5 RNA isolation

Total RNA samples were collected, using TRI® reagent (Sigma-Aldrich, St. Louis, MO, USA) according to the manufacturer's protocol, 48 and 96 hours after transfection. In brief, up to 5 × 10^6^ viable cells were harvested, re-suspended and homogenized in 0.5 mL of TRI® reagent. 0.1 mL of chloroform was added and the mixtures were centrifuged at 4°C for phase separation. The upper, aqueous phases were mixed with isopropanol and centrifuged for RNA precipitation and pelleting. The pellets were washed with 75% ethanol and then air-dried. After re-suspension in 25 μL of nuclease free water, the quantity and quality were determined by the NanoDrop ND–1000 Spectrophotometer (Thermo Scientific). Only RNA samples with a 260/280 and a 260/230 ratio of 2.0–2.1 and 1.8–2.2, respectively, were used.

### 2.6 Flow cytometry

Cells were analyzed 48 hours after transfection using the Gallios Cytometer (Beckman Coulter Inc., Brea, CA, USA). A forward/side scatter plot was used to discriminate the living from the dead cells. At least 1 ×10^4^ cells were excited by a 488 nm argon laser and the emitted signals were collected by a 525/40 BP filter.

### 2.7 Quantitation of mature miRNA levels

Mature miRNA levels were determined by quantitative real-time PCR (RT-qPCR) using the TaqMan® MicroRNA Assays (Applied Biosystems, Carlsbad, CA, USA). In general, cDNA was generated out of 10 ng total RNA in 10 μL reaction volumes via the TaqMan® MicroRNA reverse transcription kit (Applied Biosystems) according to the manufacturer's protocol. The kit includes the Multiscribe™ Reverse Transcriptase and a specific reverse-transcription primer against each miRNA. The 10 μL RT-qPCR mix consisted of the generated cDNA, the TaqMan® Universal PCR Master Mix (Applied Biosystems) and the respective 20× TaqMan MicroRNA Assay (Applied Biosystems, TM000390, TM000391, TM000524, TM000525, TM002271). Quadruplets of each cDNA sample were used for the PCR, performed on the Rotor-Gene-Q (QIAGEN). The expression levels of each mature miRNA relative to the cgr-miR-185-5p [32], an endogenous control, were determined using the 2^–ΔΔCT^ method [37]. Average fold differences in the transcript levels were determined by comparison against the negative control transfection.

### 2.8 Quantitation of primary miRNA transcripts and GFP

800 ng of DNase I (Fermentas, Waltham, MA, USA) treated total RNA of each sample were denatured for 2 minutes at 72°C and then put on ice. cDNA was generated by the DyNAmo cDNA Synthesis Kit (Thermo Scientific, Pittsburgh PA), consisting of the M-MuLV RNase H^+^ reverse transcriptase and random hexamer primers. The resulting cDNAs were diluted 1:3 and each sample was analyzed in quadruplicate RT-qPCR reactions in 10 μL with SensiMix SYBR Hi–ROX Polymerase (Bioline, UK) according to the manufacturer's protocol. Primers for the chimeric pri-miR-221 were designed to overlap the mature miRNA and the artificial flanking region of the vector. Primers for the endogenous pri-miR were designed in an analogous fashion, overlapping the stem-loop and the respective flanking regions. The RT-qPCR was performed on the Rotor-Gene Q (QIAGEN) and the transcript levels of the pri-miRNAs and of GFP relative to GAPDH were determined using the 2^–ΔΔCT^ method. Average fold differences in the transcript levels are calculated via comparison to the negative control transfection.

## 3 Results and discussion

### 3.1 Over-expression of chimeric and endogenous miRNA clusters after transient transfection

In the absence of a genomic CHO reference sequence we initially generated artificial chimeric miRNA constructs to express miRNAs in CHO cells (Fig. 1). These constructs consist of CHO-specific mature miRNAs and mmu-miR-155 loop and flanking regions that have been reported to yield high miRNA expression [26]. In order to assess the function of miRNA clusters, which are polycistronic primary miRNA transcripts that give rise to two or more mature miRNAs, we constructed two artificial miRNA cluster expression constructs (miR-15b and miR-16; miR-221 and miR-222) by sequence concatenation, as outlined in material and methods. An empty vector was used as negative control that consisted of the same expression cassette with cytomegalovirus (CMV) promoter, emGFP, but no miRNA insert in the emGFP 3' untranslated region (3'UTR). Each construct was transfected into a recombinant CHO cell line producing an Epo-Fc fusion protein (erythropoietin fused to the FC domain of immunoglobulin A) in three independent replicates. From each transfection cells were split into two batch cultures. Transfection efficiency was estimated from the portion of emGFP expressing cells 48 h after transfection (Supporting information, Fig. S1), which was previously determined to be the time point when cells reach maximum transient gene expression [32]. At this time point, 92 ± 7% of cells were GFP-positive.

**Figure 2:**
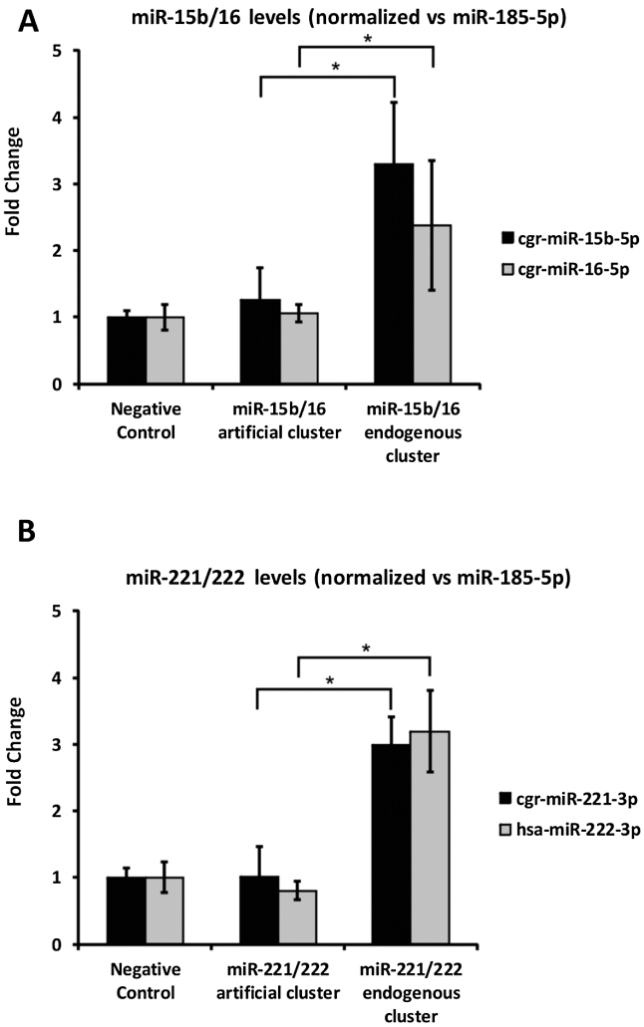
RT-qPCR analysis of mature miRNA levels. Fold changes in mature miRNA levels are shown relative to the negative control (mean ± standard deviation of three individual transfections). miR-185-5p was used as reference miRNA to assess miRNA over-expression after transfection of artificial and endogenous miRNA expression constructs. *p<0.05 (Student's t-test). **(A)** miR-15b/16-2 constructs. **(B)** miR-221/222 constructs.

The transcript levels of mature miRNA were analysed by RT-qPCR for each of the miRNAs of the two clusters (miR-15b-5p, miR-16-5p, mir-221-3p and mir-222-3p) and normalized against miR-185-5p as a stably expressed control [32]. During cDNA synthesis miRNA-specific looped RT-primers, which specifically reverse transcribe a single mature miRNA, were used to ensure amplification of mature miRNAs only. Compared to the empty vector control, the transcript levels of the mature miRNAs of the chimeric cluster constructs were not increased (Fig. 2).

Based on these results, we investigated whether the expression of miRNA clusters could be improved using the complete CHO sequence. Therefore endogenous miR-221/222 and the miR-15b/16-2 clusters were amplified from genomic DNA and cloned into the 3'UTR of the same vector that was used for the chimeric constructs (Fig. 1). The same transfection procedure as for the chimeric clusters were performed and resulted in significant 2.3 to 3.3-fold over-expression of all mature miRNAs of these clusters (Fig. 2).

### 3.2 Identification of bottleneck of chimeric miRNA biogenesis

**Figure 3:**
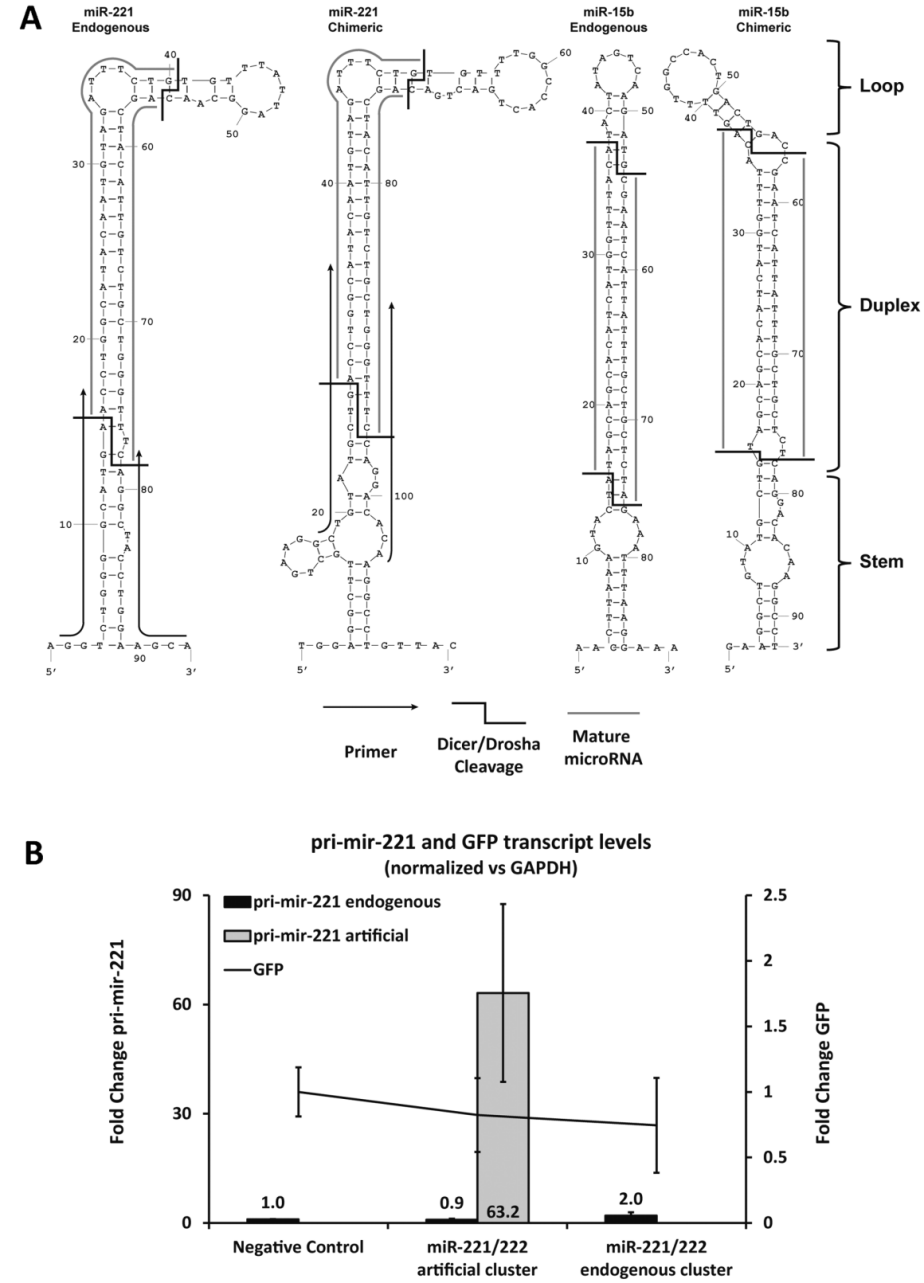
Analysis of pri-miRNA folding and transcription. **(A)** Illustration of putative secondary structures for both artificial and endogenous mir–221 and mir-15b using Quikfold [38, http://mfold.rna.albany.edu/?q=DINAMelt/Quickfold] with energy rules for RNA(3.0) and default settings. The location of primers used from amplifying the respective pri–miRNAs, the mature miR–sequences, and the cleavage sites (Drosha/Dgcr8 at the stem/duplex interface, Dicer at the duplex/loop interface) are indicated. **(B)** pri-mir-221 and GFP transcription levels two days post transfection analyzed by RT-qPCR, normalized against GAPDH and related to the negative control (mean ± standard deviation of three individual transfections).

Since emGFP expression suggested adequate transfection efficiencies and transcription rates (Supporting information, Fig. S1), and therefore availability of primary microRNA transcripts, the lack of miRNA over-expression from chimeric miRNA clusters could be due to inefficient processing in the nucleus by Drosha/Dgcr8 or in the cytosol by Dicer. To evaluate this possibility, primers were designed to amplify the primary mir-221 transcripts derived from both the endogenous and the chimeric miR-221/222 cluster (Supporting information, Table 1). These primers were designed individually for each construct, and were located at the border between mature miRNA and the flanking region (Fig. 3A, Supporting information, Table 1). RT-qPCR analysis of pri-miRNA levels after transfection of the endogenous expression construct showed a 2-fold increase in endogenous pri-miRNA levels relative to the empty vector control (Fig. 3B). This result is in line with the ∼3-fold increase observed for mature miRNA levels. However, following transfection of artifical mir-221/222 constructs, a strong (above 50-fold) increase in artificial pri-miRNA was detected when compared to the endogenous pri-miR-221 levels of the empty vector control (see Fig. 3B). This result suggests that the transcription of the chimeric miRNA clusters works well. However, possibly due to misfolding of the resulting hairpins (Fig. 3A) or to the artificial cluster sequence, the pri-miRNA transcripts are not processed and accumulate in the nucleus.

## 4 Concluding remarks

Originally, the chimeric cloning approach for vector-based miRNA expression that was used in this study was developed and tested for the stable over-expression of mouse miRNAs and shRNAs [26]. For this purpose the method is widely in use. Later, this system was adapted for use in CHO cells for single miRNAs, which yielded relatively low levels of over-expression for various mature miRNAs, ranging from 1.2 to 2.3-fold [32], depending on the overall expression level. The application of the same cloning strategy for expression of miRNA clusters in this study did not result in elevated mature miRNA levels. From our present results it appears that these constructs are not properly processed compared to constructs containing the endogenous cluster sequence amplified from gDNA. Analysis of the primary miRNA transcript level using RT-qPCR showed an enrichment of these transcripts for the chimeric constructs, suggesting that the murine flanking regions used in this study result in structural changes that cannot be efficiently processed by Drosha/DGCR8 in the nuclear processing step. Hackl et al. [34] have previously shown that while the mature miRNAs are highly conserved between human, mouse, rat, and the Chinese hamster, the homology of the hairpin sequences is much lower. In this context our results indicate that the precise secondary structure of miRNAs and, even more importantly, miRNA clusters has important implications for their processing and biogenesis. While for miRNAs and natural miRNA clusters the problem can easily be overcome using the species-specific genomic sequences for engineering purposes, it is not as easily resolved in the design of shRNAs or for construction of artificial clusters consisting of multiple miRNAs that do not naturally occur in a cluster. Here careful design of the artificial sequences taking into consideration the expected folding, especially the drosha and dicer cut sites, may be required.

## Abbreviations:

3'UTR: 3' untranslated region **CHO**, Chinese hamster ovary **CMV**, cytomegalovirus
emGFP: emerald green fluorescent protein
miRNA/miR: microRNA
pri-miRNA/pri-miR: primary microRNA **pre-miRNA**, precursor microRNA
RT-–qPCR: quantitative real time – polymerase chain reaction
RISC: RNA inducing silencing complex **shRNA**, short hairpin RNA

## References

[1] Jayapal KP, Wlaschin KF, Hu W–S, Yap MGS (2007). Recombinant Protein Therapeutics from CHO Cells - 20 Years and Counting. Chem. Eng. Prog.

[2] Krampe B, Al-Rubeai M (2010). Cell death in mammalian cell culture: molecular mechanisms and cell line engineering strategies. Cytotechnology.

[3] Trummer E, Fauland K, Seidinger S, Schriebl K (2006). Process parameter shifting: Part I. Effect of DOT, pH, and temperature on the performance of Epo-Fc expressing CHO cells cultivated in controlled batch bioreactors. Biotechnol. Bioeng.

[4] Trummer E, Fauland K, Seidinger S, Schriebl K (2006). Process parameter shifting: Part II. Biphasic cultivation – A tool for enhancing the volumetric productivity of batch processes using Epo – Fc expressing CHO cells. Biotechnol. Bioeng.

[5] Link T, Bäckström M, Graham R, Essers R (2004). Bioprocess development for the production of a recombinant MUC1 fusion protein expressed by CHO –K1 cells in protein-free medium. J. Biotechnol.

[6] Kochanowski N, Siriez G, Roosens S, Malphettes L (2011). Bmc Proc.

[7] Sung YH, Lim SW, Chung JY, Lee GM (2004). Yeast hydrolysate as a low-cost additive to serum-free medium for the production of human thrombopoietin in suspension cultures of Chinese hamster ovary cells. Appl. Microbiol. Biotechnol.

[8] Cost GJ, Freyvert Y, Vafiadis A, Santiago Y (2010). BAK and BAX deletion using zinc-finger nucleases yields apoptosis-resistant CHO cells. Biotechnol. Bioeng.

[9] Hwang SO, Lee GM (2009). Effect of Akt overexpression on programmed cell death in antibody-producing Chinese hamster ovary cells. J. Biotechnol.

[10] Majors BS, Betenbaugh MJ, Pederson NE, Chiang GG (2009). Mcl –1 overexpression leads to higher viabilities and increased production of humanized monoclonal antibody in Chinese hamster ovary cells. Biotechnol. Prog.

[11] Arden N, Betenbaugh MJ (2004). Life and death in mammalian cell culture: strategies for apoptosis inhibition. Trends Biotechnol.

[12] Borth N, Mattanovich D, Kunert R, Katinger H (2005). Effect of Increased Expression of Protein Disulfide Isomerase and Heavy Chain Binding Protein on Antibody Secretion in a Recombinant CHO Cell Line. Biotechnol. Prog.

[13] Mohan C, Lee GM (2010). Effect of inducible co-overexpression of protein disulfide isomerase and endoplasmic reticulum oxidoreductase on the specific antibody productivity of recombinant Chinese hamster ovary cells. Biotechnol. Bioeng.

[14] Mohan C, Park SH, Chung JY, Lee GM (2007). Effect of doxycycline-regulated protein disulfide isomerase expression on the specific productivity of recombinant CHO cells: Thrombopoietin and antibody. Biotechnol. Bioeng.

[15] Zhou M, Crawford Y, Ng D, Tung J (2011). Decreasing lactate level and increasing antibody production in Chinese Hamster Ovary cells (CHO) by reducing the expression of lactate dehydrogenase and pyruvate dehydrogenase kinases. J. Biotechnol.

[16] Tabuchi H, Sugiyama T (2013). Cooverexpression of alanine aminotransferase 1 in Chinese hamster ovary cells overexpressing taurine transporter further stimulates metabolism and enhances product yield. Biotechnol. Bioeng.

[17] Jeon MK, Yu DY, Lee GM (2011). Combinatorial engineering of ldh –a and bcl –2 for reducing lactate production and improving cell growth in dihydrofolate reductase-deficient Chinese hamster ovary cells. Appl. Microbiol. Biotechnol.

[18] Hackl M, Borth N, Grillari J (2012). miRNAs – pathway engineering of CHO cell factories that avoids translational burdening. Trends Biotechnol.

[19] Müller D, Katinger H, Grillari J (2008). MicroRNAs as targets for engineering of CHO cell factories. Trends Biotechnol.

[20] Barron N, Sanchez N, Kelly P, Clynes M (2011). MicroRNAs: tiny targets for engineering CHO cell phenotypes?. Biotechnol. Lett.

[21] Bartel DP (2004). MicroRNAs: genomics, biogenesis, mechanism, and function. Cell.

[22] Bartel DP (2009). MicroRNAs: target recognition and regulatory functions. Cell.

[23] Filipowicz W, Bhattacharyya SN, Sonenberg N (2008). Mechanisms of post-transcriptional regulation by microRNAs: are the answers in sight?. Nat. Rev. Genet.

[24] Winter J, Jung S, Keller S, Gregory RI (2009). Many roads to maturity: microRNA biogenesis pathways and their regulation. Nat. Cell Biol.

[25] Cullen BR (2005). Induction of stable RNA interference in mammalian cells. Gene Ther.

[26] Chung K–H, Hart CC, Al –Bassam S, Avery A (2006). Polycistronic RNA polymerase II expression vectors for RNA interference based on BIC/miR –155. Nucleic Acids Res.

[27] Ebert MS, Neilson JR, Sharp PA (2007). MicroRNA sponges: competitive inhibitors of small RNAs in mammalian cells. Nat. Methods.

[28] Otaegi G, Pollock A, Sun T (2011). An Optimized Sponge for microRNA miR –9 Affects Spinal Motor Neuron Development in vivo. Front. Neurosci.

[29] Johnson KC, Jacob NM, Nissom PM, Hackl M (2011). Conserved microRNAs in Chinese hamster ovary cell lines. Biotechnol. Bioeng.

[30] Hackl M, Jakobi T, Blom J, Doppmeier D (2011). Next-generation sequencing of the Chinese hamster ovary microRNA transcriptome: Identification, annotation and profiling of microRNAs as targets for cellular engineering. J. Biotechnol.

[31] Barron N, Kumar N, Sanchez N, Doolan P (2011). Engineering CHO cell growth and recombinant protein productivity by overexpression of miR –7. J. Biotechnol.

[32] Jadhav V, Hackl M, Hernandez Bort JA, Wieser M (2012). A screening method to assess biological effects of microRNA overexpression in Chinese hamster ovary cells. Biotechnol. Bioeng.

[33] Xu X, Nagarajan H, Lewis NE, Pan S (2011). The genomic sequence of the Chinese hamster ovary (CHO) –K1 cell line.. Nat. Biotechnol.

[34] Hackl M, Jadhav V, Jakobi T, Rupp O (2012). Computational identification of microRNA gene loci and precursor microRNA sequences in CHO cell lines. J. Biotechnol.

[35] Lattenmayer C, Loeschel M, Schriebl K, Steinfellner W (2007). Protein-free transfection of CHO host cells with an IgG-fusion protein: Selection and characterization of stable high producers and comparison to conventionally transfected clones. Biotechnol. Bioeng.

[36] Taschwer M, Hackl M, Hernandez Bort JA, Leitner C (2012). Growth, productivity and protein glycosylation in a CHO EpoFc producer cell line adapted to glutamine-free growth. J. Biotechnol.

[37] Livak KJ, Schmittgen TD (2001). Analysis of Relative Gene Expression Data Using Real-Time Quantitative PCR and the 2^–ΔΔ*C*^T Method. Methods.

[38] Zuker M (2003). Mfold web server for nucleic acid folding and hybridization prediction. Nucleic Acids Res.

