# Supplementary material for: Endogenous microRNA clusters outperform chimeric sequence clusters in Chinese hamster ovary cells
Source: Biotechnol J. 2014 Feb 12;9(4):538–44. doi: 10.1002/biot.201300216 (PMC4282078; doi:10.1002/biot.201300216)
Supplement: Supplementary file 1 [file biot0009-0538-SD1.pdf]

Biotechnology
Journal

Supporting Information for DOI 10.1002/biot.201300216

Endogenous microRNA clusters outperform chimeric sequence clusters in Chinese hamster ovary cells

Gerald Klanert, Vaibhav Jadhav, Konstantina Chanoumidou, Johannes Grillari, Nicole Borth and Matthias Hackl

© 2013 Wiley-VCH Verlag GmbH & Co. KGaA, Weinheim

Supporting Table 1 - Primer and hairpin sequences used for cloning and RT-qPCR. Mature miRNA sequences are shown in bold and italic.

Sequence ID	Application	Oligo	Sequence (5' - 3')
GAPDH	qPCR	Forward	AACTTTGGCATTGTGGAAGG
		Reverse	ACACGTTGGGGGTAGGAACA
pri-miR-221 endogenous	qPCR	Forward	AGGTCTGGGGCATGA***AC***
		Reverse	TGCTTCCAGGTAGCCT***G***
pri-miR-221 artificial	qPCR	Forward	CTGTATGCTGT***ACCTGGCA***
		Reverse	TGTGTCCTG***GAAACCCA***
miR-221/222	cluster cloning	Forward	GGAAGTCGACCTTTTCTTCCA
		Reverse	GGAACTCGAGGTCACACTCAT
miR-15b/16	cluster cloning	Forward	GGAAGTCGACGTTCTTCTGTT
		Reverse	GGAAAGATCTATGAGAGCTGCTGTATGC
chimeric miR-15b hairpin	cluster cloning		***TAGCAGCACATCATGGTTTACA***GTTTTGGCCACTGACTGAC***CGAATCATTATTTGCTGCTCT***
chimeric miR-16 hairpin	cluster cloning		***TAGCAGCACGTAAATATTGGCG***GTTTTGGCCACTGACTGAC***CCAATATTATTGTGCTGCTTTA***
chimeric miR-221 hairpin	cluster cloning		***ACCTGGCATACAATGTAGATTTCTGT***GTTTTGGCCACTGACTGAC***AGCTACATTGTCTGCTGGGTTTC***
chimeric miR-222 hairpin	cluster cloning		***TCAGTAGCCAGTGTAGATCCTG***GTTTTGGCCACTGACTGAC***AGCTACATCTGGCTACTGGGTCTCT***
GFP	qPCR	Forward	GGACGGCAACATCCTGGGGC
		Reverse	CGTCGCCGATGGGGGTGTTC

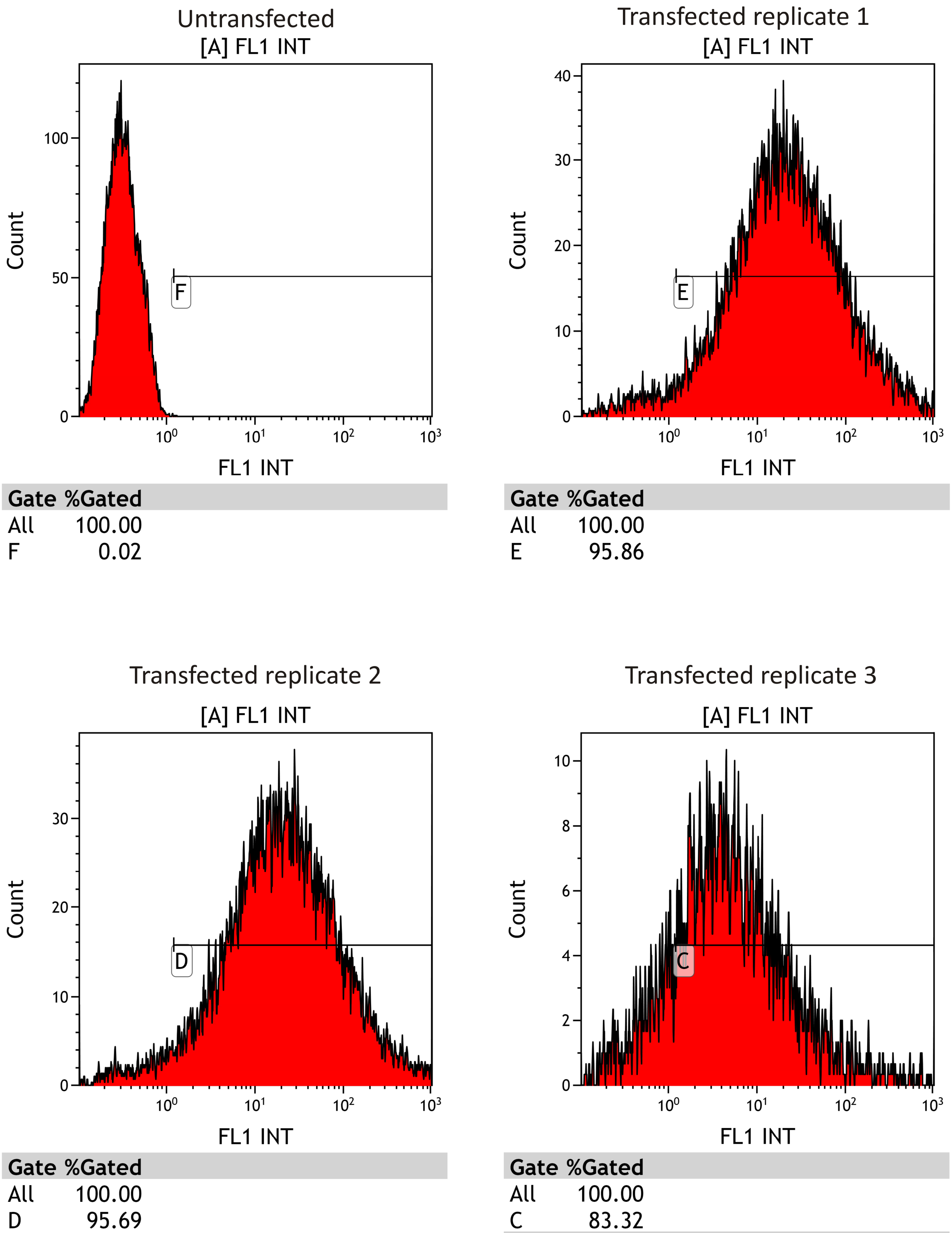

Supporting Figure 1. Transfection efficiency analysis. Flow cytometry analysis of active emGFP containing cells. GFP-expression in untransfected control cells and three replicates of cells transfected with negative control plasmid is shown after 48 h.
